# New strategies to improve results of mesh surgeries for vaginal prolapses repair – an update

**DOI:** 10.1590/S1677-5538.IBJU.2014.0163

**Published:** 2015

**Authors:** Fernando Goulart Fernandes Dias, Paulo Henrique Goulart Fernandes Dias, Alessandro Prudente, Cassio Riccetto

**Affiliations:** 1 Departamento de Urologia da Universidade de Campinas, Campinas, SP, Brasil; 2 Departamento de Urologia - Universidade Federal do Paraná, Maringá, PR, Brasil

**Keywords:** Uterine Prolapse, Surgical Procedures, Operative, Polypropylenes

## Abstract

The use of meshes has become the first option for the treatment of soft tissue disorders as hernias and stress urinary incontinence and widely used in vaginal prolapse's treatment. However, complications related to mesh issues cannot be neglected. Various strategies have been used to improve tissue integration of prosthetic meshes and reduce related complications. The aim of this review is to present the state of art of mesh innovations, presenting the whole arsenal which has been studied worldwide since composite meshes, coated meshes, collagen's derived meshes and tissue engineered prostheses, with focus on its biocompatibility and technical innovations, especially for vaginal prolapse surgery.

## INTRODUCTION

Soft tissue surgery has become the most common surgical procedure in western countries as a result of population aging as well as over-weight and obesity increasing (1). On this scenario, the use of meshes has become the first option in the treat-ment of hernia and urinary incontinence (UI) and widely used in pelvic organ prolapse (POP) treat-ment due to the high recurrence rates after primary suture techniques (2). For instance, it is supposed that about 11% of the women will undergo surgery to POP repair in their lifetime, and 30% of these patients will need reoperation because of prolapse recurrence within 4 years post-surgery (3).

POP's pathophysiology remains unclear. The strength of the pelvic floor depends on the interplay of properly innervated muscles, ligaments, and connective tissue. The etiology of prolapse is likely multifactorial, developing from obstetric trauma and denervation to the pelvic floor, as well as altered collagen and connective tissue metabolism in pelvic floor tissues (2).

Polypropylene (PP) materials became one of the most popular meshes implanted for soft tissue repair (1) due to flexibility, cellular growth and inflammatory response patterns, easy manipulation and low price (4).

Despite its popularity, complications related to mesh issues cannot be neglected, including infection and exposure of the prosthesis or erosion of vaginal wall for transvaginal implantation (5). Many of adverse effects are related to poor integration of the materials at the implantation site resulting in marked inflammation and ischemic phenomena, which delays the healing process (6). Such concerns about mesh problems in POP's treatment led the United States Food and Drug Ad-ministration (FDA) and the UK's National Institute for Health and Clinical Excellence (NICE) to publish recently warning statements regarding use of synthetic mesh for pelvic floor reconstruction based on the absence of level I evidence supporting its efficacy and safety (7).

To improve the outcome of surgical treat-ment of POP, different types of surgical implants have been launched over the last decade (8) and various strategies have been used to optimize tis-sue integration of prosthetic meshes in order to reduce complications.

The aim of this review is to perform a literature review of the state of the art on mesh's use for vaginal prolapse surgery focused on its biocompatibility and technical innovations.

## MATERIALS AND METHODS

Online searches through Pubmed, Embase and Web of Science were conducted using the search terms “mesh(es)”, “biomaterials”, “innovations“, “urinary incontinence”, “prolapses”, “biocompatibility” and “integration”. Priority was given to publications within the past 5 years in English language. Abstracts were reviewed and 58 studies were selected and subsequent suitable full texts manuscripts obtained. Of those, 7 studies were excluded for presenting data only related to hernia's treatment and 3 studies were excluded due to the lack of a reliable description of methodology. The level of evidence of each study is presented.

## MESH BIOCOMPATIBILITY

It is worthy to define mesh biocompatibility to better understand which strategies have recently been developed and tested for reducing complications and improving clinical results. It is basically determined by the foreign body reaction (FBR) and is totally connected to mesh integration.

The highly dynamic process of the FBR is considerably influenced by the biomaterial composition and its specific features. For instance, it has been well established in the literature that monofilament polypropylene mesh with large porous elicits a more biocompatible FBR resulting in better integration than multifilament and small porous size. Amid (9) devised a classification based on pore size for meshes used in hernia repairs (Table-1), which is commonly used for PP meshes description.

**Table 1:** Amid's Classification of Synthetic Biomaterials.

Type I Totally macroporous mesh with pores >75μm, the size required for infiltration of macrophages, fibroblasts, blood vessels
Type II: Totally microporous mesh with pores <10μm in at least one of three dimensions
Type III: Macroporous material with multifilamentous or microporous components
Type IV: Materials with submicronic pore size

Ref: 9-Amid PK: Classification of biomaterials and their related complications in abdominal wall hernia surgery. Hernia. 1997;1:15-2.

Graft options have evolved over years, and include autografts, allografts, xenografts and synthetic materials (Table-2). Surgeon preference for graft material has varied widely, and for each material its own inherent advantages and disadvantages have been described (10).

**Table 2:** Biomaterials Options For Soft Tissue Repair.

Natural	Synthetic
Autografts (rectus fascia, fascia lata, vaginal wall);	Absorbable: Polyglactic acid Permanent:
Allografts (cadaveric tissues, including dura mater, dermis, fascia lata);	Polytetrafluoroethylene; Polypropylene; Polyvinylidene fluoride;
Xenografts (porcine small intestinal submucosa, porcine dermis)	Silicone elastomers; Polyester

Modifications of the polymer's chemical composition, material weight, filament structure and pore size have substantial effects in the in vivo biocompatibility and are potential parameters to be studied in order to improve clinical results.

## RESEARCHES ON NEW POP PROSTHESIS

We have didactically divided researches into 5 topics that will further be presented:

- Composite Meshes
- Changes In Mesh Density And Pore Sizes
- Coating Biomaterials
- Collagen Derived Biomaterials
- Tissue Engineered Protheses (TEP)

## A. COMPOSITE MESHES

Theoretically, the rationale of using a composite mesh is to associate an absorbable component, which will reduce the amount of foreign material in the host without compromising the mechanical resistance in the end of the integration process. Moreover, absorbable components can act as a long lasting dressing during healing period (11, 12). Recently, many combinations have been tested (PP+polyglactin; PP+polysaccharide hydrogel) with different Levels of Evidence (LE).

### 1. PP+Polyglactin (PG)

Clinical studies using polyglactin have already been published. In 2001 Weber et al. (13) compared three techniques of anterior colporrhaphy: standard; standard plus polyglactin 910 mesh; or ultralateral anterior colporrhaphy, and concluded that the addition of polyglactin 910 mesh did not improve the cure rate compared with standard anterior colporrhaphy and provided similar anatomic cure rates and symptom resolution for anterior vaginal prolapse repair. In 2005, an observational study involving 90 patients was conducted with retrospective chart review and prospective subjective and objective assessments at the end of a 1-year study period to access the results of colporrhaphy with a composite vicryl-polypropylene mesh. Surgical correction was achieved in 27 of 31 (83.9%) at 6 months and beyond and there was no mesh infection but minor vaginal mesh protrusion was found in 7 of 90 (7.8%) patients at 6-12 weeks and 4 of 31 (12.9%) patients at 6 months and beyond. The conclusion was that posterior colporrhaphy with mesh was effective in treating posterior vaginal prolapse (14).

Experimental studies about this subject are also available. Junge et al., in an experimental study using 60 male Sprague-Dawley rats, found benefits of using a partially absorbable (PG+PP) mesh in terms of a reduced amount of biomaterial after implantation and absorption of the absorbable part when compared with a standard non––absorbable PP mesh (15) (LE: 5). However, some studies found an increased inflammation induced by polyglactin (13, 14). After implantation in a standardized rodent animal model, The Vypro II (PP+PG) presented with increased inflammatory and fibrotic reaction in comparison with PP group (16) (LE: 5). Also, Weyhe et al. (17) described increased levels of IL-6 and suppressed levels of transforming growth factor-beta 1 in the composite mesh group, using an NRK-49F (mixed culture of normal rat kidney cells) fibroblast culture.

### 2. PP+Polysaccharide Hydrogel

A hybrid mesh (HM) composed of polypropylene mesh embedded in a polysaccharide hydrogel was studied after intramuscular and subcutaneous implantation in rats in comparison with two clinically used materials. Histological and mechanical aspects were analyzed and the authors concluded that HM's use is promising because it is easy to make, easily implanted, and once inserted produces little foreign body reaction, creating a well-balanced integration with the tissue environment. Further, using the properties of this hydrogel to deliver substances locally (growth factors, antibiotics, etc.) can be used to regulate tissue colonization (18) (LE: 5).

## B. CHANGES IN MESH DENSITY AND PORE SIZES

Theoretically, low-density meshes would optimize the FBR by reducing the mass of the material used and diminishing its contact interface with the host tissue (19). However, there are studies with conflicting results regarding the real advances brought in by low-density meshes. Klinge et al. (20) examined meshes with different pore sizes and weight in a rat abdominal model and found that lightweight meshes had superior tissue integration, reduced inflammation, and reduced fibrosis compared with the heavyweight mesh, which corresponded to a reduction in cell turnover in the lightweight meshes (LE: 5). In a review, Weyhe et al. (21) concluded that light meshes seemed to present certain advantages in relation to postoperative pain and the foreign-body feeling, but were associated with a greater recurrence rate (LE: 1A).

## C. COATING BIOMATERIALS

Biomaterial surface properties play an important role. The use of some agents for coating biomaterials is based on their ability to mask the underlying surface by producing a hydrophilic interface. These coating layers improve the device/ host tissue interactions and consequently improve device functionality and life span (22, 23). Various materials are currently used for a device coating, including natural or synthetic polymeric materials (24) (Table-3), each one of these with a particular purpose, such as antiadhesive, infection protection and protection against foreign body reaction.

**Table 3:** Options for Synthetic Device Coating.

Natural		References
	alginate	53-54
	chitosan	55
	collagen	56-57
	dextran	58-59
**Synthetic**		
	poly-lactic-acid	60-61
	poly-lacticco-glycolic-acid (PLGA)	62
	poly-ethylene-glycol (PEG)	63
	poly-vinyl-alcohol (PVA)	64-65

### 1. Corticosteroids Coating

As therapeutic drugs, corticosteroids have been used for their immunosuppressive benefits in daily practice for decades. Recent emphasis on their effects on tissue remodeling and fibrosis in FBR underline their therapeutic potential (25). Brandt et al. developed a mouse model to investigate the role of the mineralocorticoid and glucocorticoid's receptors in FBR around steroid-coated polyvinylidenfluoride (PVDF) meshes. Early modification of corticosteroid receptor activity can attenuate local inflammatory response to foreign bodies with subsequent beneficial long-term effects. Further investigations should clarify dose response, the protective potential of a combination of both protective substances, and whether similar effects could be achieved by systemic delivery of drugs (26) (LE: 5).

### 2. Plasma

Animal experiments support the hypothesis that autologous modification of alloplastic materials by coating with plasma leads can improve early integration of the respective material into the different locations. Gerullis et al. (27) implanted coated and uncoated version of three different mesh types, all of them with previously in vitro proved biocompatibility. They demonstrated significant less FBR, scar formation and inflammatory reaction for the plasma coated material in each type of mesh after 3 and 6 months with the following ranking order: (1) Dynamesh-CICAT™; (2) Ultrapro™; (3) TVTO™ (LE: 2). Accordingly, it was found an improved cell adhesion on alloplastic meshes covered with autologous plasma compared to non-covered meshes including eighteen different commonly used alloplastic materials (28).

### 3. Titanium-Coated Mesh

Titanium is considered to be an inert material and has been used for decades in orthopedic and dental implants. As a coating, it is thought to resist to degradation at room temperature due to a thin and stable protective oxide layer that forms on its surface.

Titanium-coated PP meshes have more recently come into use in the field of pelvic re-constructive surgery and hernia repair. However, studies of titanized meshes used for hernia repair in animal models have not clearly shown any superior benefit, with no significant differences (29) or even greater inflammation (15) (LE: 5). Moreover, titanium-coated polypropylene meshes have no particular advantages in infected tissue (30) and safety's concern still needs to be clarified (31) (LE: 5).

### 4. PP+Poly(Vinyl Alcohol)

**Figure 1:**
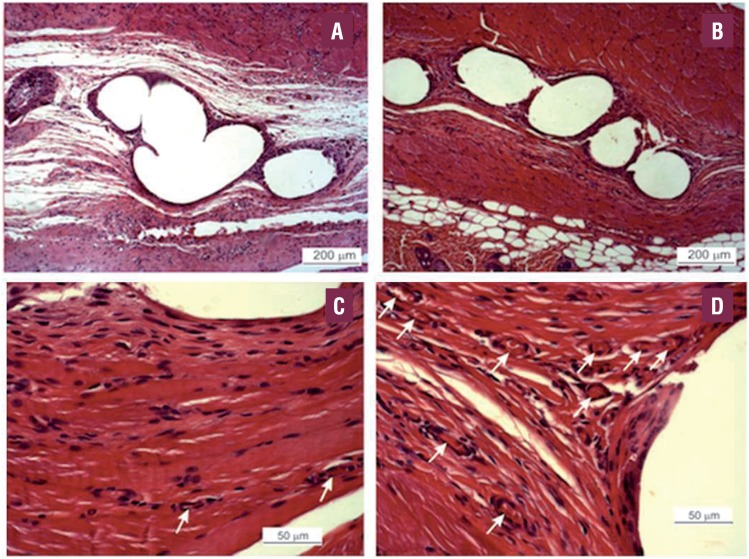
Composite Mesh: PVA/GSNO-40-impregnated PP mesh. Representative micrograph of the subcutaneous tissue surrounding filaments of PP meshes 21 days after implantation in the subcutaneous tissue of Wistar rats. A) HE 100×; Plain PP mesh (control). B) HE 100×; PVA/GSNO-40-impregnated PP mesh. C) HE 400×. Plain PP mesh. D) HE 400×; PVA/GSNO40-impregnated PP mesh. Note the presence of fewer spaces among collagen fibers around the filaments in (B) which means lower edema; Note the higher amount of vessels (arrows) in (D) which means greater angiogenesis.

Prudente et al. (32) impregnated mono-filament PP meshes with physically cross-linked poly (vinyl alcohol) (PVA). The PVA deposits were also used as reservoirs for the local release of S––nitrosoglutathione (GSNO), a nitric oxide (NO) donor. Histological analysis of the abdominal wall, 21 days after the implant in rats, revealed lower edema (p=0.0039) and greater angiogenesis (p=0.0031), based on immunehistochemical expression of CD-31 surface antigen surrounding the implant (LE: 5). A marked decrease of NO concentration in the tissue surrounding the impregnated meshes was observed after 2 days (Figure-1) and authors concluded that improvements in FBR were due to hydrophilicity changes in the local micro-environment around the mesh.

(Figure is a courtesy of author: Prudente el al., 2013).

### 5. Antibiotics

An anti-infective drug-eluting mesh that slowly delivers antibiotic around the surgical site could be used to avoid bacterial contamination of the prosthesis and subsequent biofilm formation. The local delivery system offer several advantages over systemic administration: greater efficacy, lower drug dose required, less toxicity due to the lo-cal release of the drug, extended activity and less likelihood of promoting antimicrobial resistance (33, 34) (LE: 1A). A new antibiotic-eluting system was developed on a pre-existing type IPP mesh used for the treatment of genital prolapse, without having an impact on the intrinsic properties of the material, and proved to decrease post-operative short-term infection (1) (LE: 3).

### 6. Silver-Coated Mesh

For many years, silver has been a precious metal that is used in several fields of medicine and surgery for its anti-infectious properties (35). A study compared silver-coated and non-silver-coated large pore monofilament PP mesh implants with and without infection (four groups) inserted in the abdominal wall of 84 female Wistar rats. An Escherichia coli strain was inoculated intraoperatively in the two infected groups. The implants were removed, and clinical, bacteriological, and histological analysis were performed at 2, 15, and 30 days postoperatively. All inoculated rats (n=21) in the non-silver-coated PP group presented periprosthetic E. coli infection, compared with only five inoculated rats in the silver-coated PP group (p<0.0001). Erosion was significantly higher in the infected than in the non-infected silver-coated PP groups (p<0.01). There was no histological difference between the four groups. They concluded that silver-coated implants appear effective against bacterial infection, with good histological tolerance but delayed healing (LE: 5). However, the potential cytotoxicity of silver, as well as bio-mechanical properties of polypropylene after the release of silver nanoparticles, require further studies (36).

## D. COLLAGEN DERIVED BIOMATERIALS

Collagen-based biomaterials have been available for several decades and are becoming increasingly popular due to their perceived biocompatibility and low immunogenicity (37).

### 1. Collagen Coating

Collagen type I has been used as a coating for polypropylene mesh because of its unique bio-logical properties. It is the most abundant protein in the mammalian connective tissues. Its structure is very conservative and therefore it does not induce an immune reaction even if a xenogeneic material is used. It enhances and augments growth of mesothelial cells that act as an antiadhesive layer (38). Some reports have confirmed the utility of collagen foil as such a barrier (39).

Concerning urogynecology surgeries, some interesting studies are available. Huffaker et al. (40) used a rabbit vagina model to compare tissue responses to two PP meshes: uncoated PP mesh (Gynemesh PS^TM^) versus collagen-coated PP mesh (Pelvitex^TM^) at 12 weeks and found similar scores for inflammation, neovascularization, and fibroblastic proliferation, but a higher apoptotic activity in the collagen-coated group (0.39% vs.0.1%; p: 0.04) (LE: 5). De Tayrac et al. used a sheep vagina model (41), and concluded that a delayed tissue integration was observed in the collagen-coated mesh one week after implant, despite a non-significant higher rate of vaginal erosions in the uncoated group (33% vs. 6.7%; p:0.4) (LE: 5). Other authors also suggest that use of a collagen––coated PP mesh for vaginal prolapse repair may help reduce erosions and dyspareunia (42) (LE: 3) and the mechanism involved could be due to the lesser adhesion of the coated mesh on the vaginal wound during the early postoperative period. On the other hand, reports on increased susceptibility to infections and lack of long-term effectiveness of such a coating have been published (43) (LE: 2).

**Figure 2:**
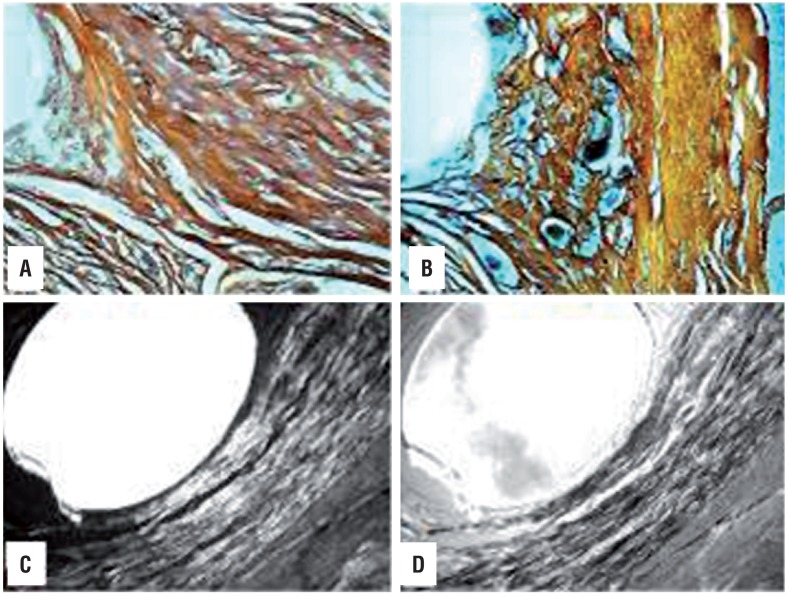
Highly Purified Collagen Gel Coating Polypropilene Mesh presenting higher birefringence level of collagen fibers, thus reflecting an improved molecular organization of newly formed collagen. A and B) Image of section impregnated with silver (Ag) 90 days after implantation. PP (A) and PP+C (B) where the packing arrangement of collagen fibers is demonstrated. Ag, 40×1. C and D) Unstained section immersed in water (PP+C 90 days after implantation), showing filament cut perpendicularly, through collagen fibers with various brightness intensities. Birefringence is revealed by brightness contrasted with a dark background. B. Same image, after compensation, in which collagen fibers appear dark.

In an experimental study, PP mesh coating with a new highly purified collagen gel consisting of type I collagen obtained from bovine tendon was implanted in a rat model and showed an in-crease of adherence of the mesh to the neighboring tissue, less intense and persistent lymphocyte, plasma cell, and granulomatous reaction and a higher birefringence level of the collagen fibers, thus reflecting an improved molecular organization of newly formed collagen and a positive re-modeling action in mesenchymal repair involving polypropylene mesh (44) (LE: 5) (Figure-2).

(Figure is a courtesy of author: Siniscalchi et al., 2013).

### 2. Porcine Small Intestinal Submucosa (SIS)

Porcine small intestinal submucosa (SIS) is an almost acellular biomaterial, which is derived from the submucosal layer of pig small intestine and exhibits several characteristics that are advantageous for sling placement: its extracellular matrix promotes ingrowth of cells that remodel it into functional host tissues; its cytokines attract host cells and promote angiogenesis; it is strong, easily sutured, and can be easily manufactured to match the physical properties of the tissue being replaced; and elicits only a mild immunologic response (45). In a comparative study between PP and non-cross-linked porcine small intestine serosal-derived collagen implants in a rat model, SIS induced a less pronounced inflammatory re-action, less dense adhesions and an architecturally better collagen organization than PP. SIS shows an obvious transient weakness in tensile strength at 30 days compared with PP but this difference does not persist (46) (LE: 5).

## E. TISSUE ENGINEERED PROTHESES (TEP)

Recent advances in cell-based technology using regenerative medicine techniques suggest that this approach holds enormous potential to improve human conditions by encompassing alteration of the current biological state of a targeted tissue, augmentation of depleted function, or absolute functional tissue replacement (47). To that end, numerous cell-based investigations have been performed to address urinary incontinence. Cells derived from various sources have been used for urinary incontinence, including chondrocytes, smooth muscle cells, muscle precursor cells, adipose-derived stem cells, and bone marrow stromal cells providing coaptation of the bladder neck by augmenting tissue mass or restoring sphincter function (48).

The potential use of cell-based tissue engineering strategies to treat POP appears to be more intricate. The vagina is a complex organ with great demands of functionality, and parameters such as strength and elasticity of the native tissue vary interpersonally (49). Furthermore, the pathological anatomy of POP dictates that a simple injection of cells to regenerate damaged vaginal tissue is not feasible. Besides these limitations, a novel tissue engineering approach for creating prostheses for the treatment of stress urinary incontinence and pelvic organ prolapse has been described. TEP were made from stromal fibroblasts, obtained from oral mucosal biopsies, cultured in Dulbecco's Modified Eagle's medium (DMEM) on an electrospun fabric of Poly(L)lacticco glycolic acid (PLGA) polymer. This technique allows for ingrowth of cells with the intrinsic ability to produce collagen. So far, only limited evidence exists, and additional animal studies are imperative before this approach is ready for clinical use (50).

## DISCUSSION

This review was based on a compilation of selected studies concerning mesh and biomaterials innovations using Pubmed, Embase and Web of Science databases. It is important to emphasize the lack of good quality clinical studies and the great difficult to compare them due to different methodologies. Moreover, the majority of studies are experimental and current literature does not have a reliable animal model for stress urinary incontinence and/or prolapse repair. Although it has already been tested by numerous previous experiments to evaluate graft and prosthesis integration, the rat subcutaneous tissue surely is not the ideal experimental model. In current literature different models have been tested with interesting findings, including rabbit and sheep's vagina models (40, 41). Thus, those results cannot just be extrapolated to clinical practice. However, despite this weakness, such review can be useful as a brief exposition of mesh innovations and strategies under development for biocompatibility improvement (Table-4).

**Table 4:** Biomaterials for Reconstructive Surgery-Innovations.

**Composite Meshes**	
	Polypropylene + Polyglactin
	Polypropylene + Polysaccharide hydrogel
**Coated Meshes**	
	Antiadhesive
	Infection protection (Antibiotics, Silver)
	Foreign Body Reaction (Corticosteroids, Plasma, Titanium, Collagen, Polyvinyl alcohol)
**Collagen Based Meshes**	
	Bovine
	Porcine (SIS)
**Tissue Engineered Meshes**	

The major controversy is still the use of meshes for pelvic organ prolapse repair. The literature does not have strong evidences, which support their use for that purpose as a routine. Therefore, continuous research is needed in order to find an ideal biomaterial, with an adequate size, weight and features for lowering the risk of infection and integration defects. Moreover, as concluded by Barski et al. in a meta-analysis, careful individualized selection of patients and materials, education of patients, and elimination of identified risk factors are urgent prior to implantation of vaginal meshes (51).

The vagina is considered a clean-contaminated field. Mesh complications, such as erosion, may be linked to bacterial contamination at the time of mesh insertion (2). The properties of the mesh play an important role in lowering the infection risk. The major concern is related to vaginal prolapse surgery, in which the size of the meshes and positioning in the vagina differs substantially from the sub-urethral tapes (52).

### Comments on each topic

#### A. Composite Meshes

Recently, many combinations of composite meshes have been tested (PP+olyglactin; PP+polysaccharide hydrogel; PP+polyvinyl alcohol) but with conflicting results. It is important to state that some evidences are based on experimental studies without an adequate control, and with a short follow-up (18). Moreover, some conclusions were based on in vitro studies (15–17).

#### B. Changes In Mesh Density And Pore Sizes

Concerning the ideal mesh weight, light or ultralight meshes tend to become first choice, because of its better biocompatibility, but still needs further improvements to avoid the risk of higher recurrence rate.

#### C. Coating Biomaterials

Coating agents seems to be a great potential strategy, highlighting antibiotic and corticosteroid coating with interesting preliminary in vitro results. Silver and titanium's use should have its cytotoxicity and biomechanical aspects clarified.

#### D. Collagen Derived Biomaterials

Due to its availability, biocompatibility, low immunogenicity and favorable biological properties (37), collagen is an imminent candidate on biomaterial's scenario. However, just few trials are available with different collagen preparations. As other biological products, there is a wide range of purification, decellularization and cross-linked treatments among collagen preparations presented, making comparisons difficult.

#### E. Tissue Engineered Protheses (TEP)

Cell-based technology will surely become one of the most relevant approaches for soft tissue disorders in a few years. However, so far, only limited evidence exists and, especially considering POP's treatment, cell-based tissue engineering is still just speculation.

## CONCLUSIONS

Biomaterials research have recently emerged worldwide as a result of an urgent need for more appropriate options for reconstructive medicine and treatment of soft tissue disorders. Composite or coated meshes intending to get antiadhesive profile, protection against infection or to elicit less pronounced foreign body reaction have been tested. Although significative basic science information has been produced, there are still conflicting results. Also, tissue-engineering techniques have shown only limited evidence till now. As the life expectancy is growing quickly, efforts in biomaterials research must be reached, in order to offer long lasting therapeutic options for re-constructive surgery.
